# Killer cell immunoglobulin-like receptor (KIR) gene content variation in the HGDP-CEPH populations

**DOI:** 10.1007/s00251-012-0629-x

**Published:** 2012-07-01

**Authors:** Jill A. Hollenbach, Isobel Nocedal, Martha B. Ladner, Richard M. Single, Elizabeth A. Trachtenberg

**Affiliations:** 1 Center for Genetics, Children’s Hospital Oakland Research Institute, Oakland, CA 94609 USA; 2 Department of Mathematics and Statistics, University of Vermont, Burlington, VT 05405 USA

**Keywords:** Killer cell immunoglobulin-like receptor, KIR, HGDP-CEPH, Population, Diversity

## Abstract

In the present study, we investigate patterns of variation in the *KIR* cluster in a large and well-characterized sample of worldwide human populations in the Human Genome Diversity Project—Centre d'Etude du Polymorphisme Humain (HGDP-CEPH) panel in order to better understand the patterns of diversity in the region. Comparison of *KIR* data with that from other genomic regions allows control for strictly demographic factors; over 500,000 additional genomic markers have been typed in this panel by other investigators and the data made publicly available. Presence/absence frequencies and haplotypic associations for the *KIR* region are analyzed in the 52 populations comprising the panel and in accordance with major world regions (Africa, Middle East, Central Asia, East Asia, Europe, Americas, and Oceania). These data represent the first overview of *KIR* population genetics in the well-documented HGDP-CEPH panel and suggest different evolutionary histories and recent selection in the *KIR* gene cluster.

**Electronic supplementary material:**

The online version of this article (doi:10.1007/s00251-012-0629-x) contains supplementary material, which is available to authorized users.

## Introduction

The killer cell immunoglobulin-like receptors (*KIR*) (Vilches and Parham 2002) are a family of receptors expressed on natural killer (NK) cells and a small percentage of cytotoxic T cells used to regulate cell killing and cytokine response (Biron 1997; Vales-Gomez et al. 1998; Bashirova et al. 2006; Young and Uhrberg 2002; Hsu 2004; Smyth et al. 2005). The *KIR* act to inhibit or activate NK cells, and while the inhibitory *KIR* use the human leukocyte antigen (HLA) class I molecules as their ligand, the ligands for most of the stimulatory *KIR* have not been identified definitively. The balance between inhibitory and activating *KIR* and their specific ligands results in a finely tuned innate-adaptive immune response. The inhibitory *KIR*, partnered with alternative NK receptor complexes (CD94:NKG2A), ensure that NK cells are tolerant of healthy autologous cells and responsive to cells with compromised HLA class I expression, as what occurs frequently in virus-infected and tumor cells. The stimulatory *KIR* contribute to the activation of NK cells in response to infection and malignancy. Our recent work and that of others has shown that *KIR* and their HLA ligands are associated in a variety of autoimmune diseases (Parham 2005; Khakoo and Carrington 2006; Williams et al. 2005; Hollenbach et al. 2009) and infectious diseases (HIV and hepatitis C; Martin et al. 2007; Li et al. 2004; Khakoo et al. 2004) as well as being important in solid organ and hematopoietic stem cell transplant (Cooley et al. 2009, 2010; Ruggeri et al. 2002; Kunert et al. 2007) and pregnancy (Moffett and Hiby 2007; Moffett-King 2002; Lanier 1999).

**Fig. 1:**
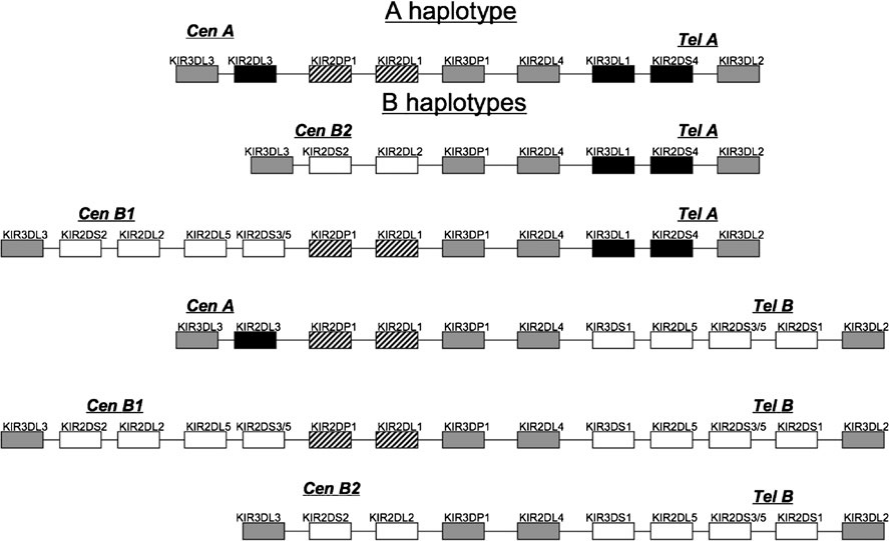
Structure of the six most common KIR gene content haplotypes in most of the world populations. The centromeric (*Cen*) and telomeric (*Tel*) motifs are labeled according to their association with the KIR A or B haplotype

The *KIR* gene complex is located on human chromosome 19q13.4 and is both polygenic and extremely polymorphic; further variation at the functional level is derived from the variegated expression pattern of the KIR gene products on the surface of NK cells (Young and Uhrberg 2002). While extensive allelic variability has been identified, particularly in the inhibitory genes (http://www.ebi.ac.uk/cgi-bin/ipd/kir), variability in gene content haplotypes is responsible for significant diversity both within and between populations. While many *KIR* haplotypes have been identified in human populations, they are generally categorized into two groups, A and B (Martin et al. 2004; Parham 2005); the A haplotype is represented by a single configuration of mainly inhibitory genes, while numerous possible B haplotypes are typified by varying numbers of additional activating genes (Middleton et al. 2007b; Uhrberg et al. 2002; Hsu et al. 2002). Six gene content haplotypes (Fig. 1) that have been identified through segregation (Khakoo and Carrington 2006) and sequence analysis (Pyo et al. 2010) appear to be relatively common across several major ethnic groups, accounting for greater than 90 % of the *KIR* haplotypic variation observed.

Previous work has suggested that very little linkage disequilibrium (LD) exists between the centromeric and telomeric sections of the *KIR* cluster, while there is a strong LD within these regions (Gourraud et al. 2010). The six *KIR* haplotypes in Fig. 1 are all combinations of three centromeric and two telomeric basic motifs (Pyo et al. 2010). These motifs are associated (and named in accordance) either with the canonical *KIR* A inhibitory haplotype, or variations of the more stimulatory *KIR* B haplotypes. Four framework genes are found in almost all *KIR* haplotypes and flank the Cen and Tel motifs, including the centromerically located *KIR*3DL3 and *KIR*3DP1, and the telomerically located *KIR*2DL4 and *KIR*3DL2 loci*.* Centromerically, Cen-A is characterized by the presence of *KIR*2DL3, *KIR*2DP1, and *KIR*2DL1. Cen-B1 has the longest motif characterized by the presence of *KIR*2DL2, *KIR*2DS2, *KIR*2DL5, *KIR*2DS3S5, *KIR*2DP1, and *KIR*2DL1. The truncated Cen-B2 motif has only *KIR*2DL2 and *KIR*2DS2. Telomeric motifs are Tel-A, characterized by the presence of *KIR*3DL1 and *KIR*2DS4, and Tel-B, characterized by inclusion of *KIR*3DS1, *KIR*2DL5, *KIR*2DS3S5, and *KIR*2DS1. A seventh haplotype identified by Pyo et al. (2010) consists of a fourth centromeric motif (Cen-B3) with Tel-A; the Cen-B3 motif appears to be midway between Cen-B1 and Cen-A, with *KIR*2DL3, *KIR*2DL5, *KIR*2DS3S5, *KIR*2DP1, and *KIR*2DL1, and has been observed only at low frequencies in reference populations.

The apparent rapid evolution within the *KIR* complex via repeated recombination and duplication events (Khakoo et al. 2000; Vilches and Parham 2002; Guethlein et al. 2007; Canavez et al. 2001), coupled with the critical role of natural killers cells in innate immune surveillance, suggests that pathogen-driven selection may in part drive the worldwide patterns of genetic variation observed in the *KIR*, as has been proposed for the *HLA* region (Trowsdale and Parham 2004; Trowsdale 2001; Vilches and Parham 2002)*.* The rapid evolution of the *KIR* relative to the *HLA* genes may be reflective of selective processes promoting more efficient interactions with HLA class I molecules (Khakoo et al. 2000). Further selective pressures are likely related to the interaction of *KIR* and *HLA* genes in reproduction (Hiby et al. 2004; Moffett-King 2002). Our recent work has documented worldwide diversity in the *KIR* cluster (Hollenbach et al. 2010) and supports the notion that *KIR* are co-evolving with *HLA* (Single et al. 2007b). Like *HLA*, the *KIR* repertoire is likely to have been shaped by numerous events in human population history, most notably migrations to, and subsequent expansions within, new geographic and climactic environments. Evolutionary pressures stemming from the transition from hunter-gatherer to agrarian societies; the sporadic, abrupt introduction of new pathogens to populations from outside sources; and the continued variation in pathogenic type and load at the local level may all have played a role in shaping the *KIR* repertoire globally. While the available data related to *KIR* variation in human populations have increased in recent years, the challenge in studies of this nature is to disentangle variation related to selective events from those tied to population history (Meyer et al. 2006; Single et al. 2008).

Interest in the characterization of worldwide *KIR* diversity is ongoing, with published complete gene content profiles for over 100 populations to date, and many more populations typed for one or a few *KIR* loci (allelefrequencies.net). However, direct comparisons between populations are hindered by a lack of equivalence across data sets due to variations in typing methodology and the number of loci typed, as well as clarity regarding population collection and limited data regarding other genomic markers. In the present study, we investigate patterns of variation in the *KIR* cluster in a large and well-characterized sample of worldwide human populations in the Human Genome Diversity Project—Centre d'Etude du Polymorphisme Humain (HGDP-CEPH) panel (Cann et al. 2002) in order to obtain a comprehensive overview of diversity in the *KIR* gene cluster. Over 500,000 additional genomic markers have been typed in this panel by other investigators and the data made publicly available; comparison of *KIR* data with that from other genomic regions allows control for strictly demographic factors. These data represent the first overview of *KIR* population genetics in the well-documented HGDP-CEPH panel, and in publishing complete *KIR* genotypes and carrier frequencies substantially increases the available population level data for this important gene system.

## Materials and methods

### Subjects

The 52 populations comprising the HGDP-CEPH represent seven major world regions. *KIR* typing was performed using genomic DNA from 976 individuals from this panel after removal of atypical samples and related individuals (Cann et al. 2002). Sample sizes range for these populations between 7 and 46 individuals, with an average of 19 individuals (38 chromosomes) per population. Population details are given in Table 1. Populations are grouped by world regions as given in the HGDP-CEPH Stanford database (http://spsmart.cesga.es/ceph.php?dataSet=ceph_stanford).

**Table 1:** HGDP-CEPH populations, sample size, and geographic location

Population	Number	Region	Latitude	Longitude
Bantu N.E.	11	Africa	3 S	37 E
Bantu S	8	Africa	29 S	30 E
Biaka Pygmies	29	Africa	30–31	66–67 E
Mandenka	23	Africa	12 N	12 W
Mbuti Pygmies	12	Africa	1 N	29 E
San	7	Africa	21 S	20 E
Yoruba	23	Africa	6–10 N	2–8 E
Mozabite	27	Middle East	32 N	3 E
Bedouin	46	Middle East	4 N	17 E
Palestinian	42	Middle East	32 N	35 E
Druze	46	Middle East	32 N	35 E
Adygei	17	Europe	44 N	39 E
French	26	Europe	46 N	2 E
French Basque	22	Europe	43 N	0
North Italian	14	Europe	46 N	10 E
Orcadian	13	Europe	59 N	3 W
Russian	24	Europe	61 N	39–41 E
Sardinian	24	Europe	40 N	9 E
Tuscan	8	Europe	43 N	11 E
Pathan	21	Central-South Asia	32–35 N 69	72 E
Makrani	25	Central-South Asia	26 N	62–66 E
Kalash	24	Central-South Asia	35–37 N	71–72 E
Hazara	22	Central-South Asia	33–34 N	70 E
Balochi	23	Central-South Asia	30–31 N	66–67 E
Barusho	20	Central-South Asia	31 N	35 E
Brahui	22	Central-South Asia	36–37 N	73–75 E
Sindhi	23	Central-South Asia	24–27 N	68–70 E
Uygur	9	Central-South Asia	44 N	81 E
Cambodian	11	East Asia	12 N	105 E
Dai	8	East Asia	21 N	100 E
Daur	9	East Asia	48–49 N	124 E
Han	46	East Asia	36–39 N	108–120 E
Hezhen	10	East Asia	47–48 N	132–135 E
Japanese	31	East Asia	38 N	138 E
Lahu	10	East Asia	22 N	100 E
Miaozu	8	East Asia	28 N	109 E
Mongola	10	East Asia	45 N	111 E
Naxi	10	East Asia	26 N	100 E
Orogen	9	East Asia	48–53 N	122–131 E
She	10	East Asia	27 N	119 E
Tu	9	East Asia	36 N	101 E
Tujia	7	East Asia	29 N	109 E
Xibo	8	East Asia	43–44 N	81–82 E
Yakut	22	East Asia	62–64 N	129–130 E
Yizu	10	East Asia	28 N	103 E
Papuan	16	Oceania	4 S	143 E
NAN Melanesian	21	Oceania	6 S	155 E
Karitiana	24	America	10 S	63 W
Maya	24	America	19 N	91 W
Pima	25	America	29 N	108 W
Surui	15	America	11 S	62 W
Colombian	12	America	3 N	68 W

### KIR genotyping

To genotype the *KIR* loci in the HGDP-CEPH, we utilized our high throughput single nucleotide polymorphism (SNP)-based *KIR* genotyping assay developed using the SEQUENOM^™^ MassARRAY (San Diego, CA, USA) on the matrix-assisted laser desorption/ionization time-of-flight (MALDI-TOF) mass spectrometer platform (Houtchens et al. 2007), which we modified to improve efficiency and accuracy, including all known alleles at the time (Hollenbach et al. 2009). The assay types for the presence or absence of 14 *KIR l*oci and common alleles, including *KIR*2DL1*004, *KIR*2DS4*003/004/006/007 (form with truncated protein products which are not expressed on the cell surface; Middleton et al. 2007a), and *KIR*2DS4*001 (expressed and capable of being membrane-bound). Briefly, the two-tiered analysis includes 38 “capture” primer pairs to amplify specific *KIR* genes in the region surrounding the SNPs to be queried; this is followed by 39 homogenous mass extend primer reactions to differentiate individual SNP patterns for the 16 *KIR* genes on the MALDI-TOF platform. These assays were run using the *KIR* sequence alignment in the Immuno Polymorphism Database (IPD; http://www.ebi.ac.uk/ipd/kir) using version 1.4.0. Our typing system is regularly assessed against all known alleles in order to insure that all loci will be detected.

### Statistical analysis and data visualization

A treemap of HGDP-CEPH sample size and number of unique KIR genotypes was constructed using the “tmPlot” function in the “Treemap” package (Tennekes 2010) for the R language for statistical computing (R Development Core Team 2008). The base code for the “dens2col” function was modified to produce a grayscale image, otherwise default settings for the “dens” option were used.

Carrier frequencies for the *KIR* loci were obtained by direct counting. Gene frequencies were estimated according to Lynch and Milligan (1994) using the “frequency” function for binary data in a diploid population in the GenAlEx package (Peakall and Smouse 2006). For the locus *KIR*2DL2L3 (*KIR*2DL2/KIR2DL3), gene frequencies were obtained by direct counting. A direct counting approach was also utilized to obtain an estimate for gene frequencies for the *KIR*3DL1S1 locus. There is evidence that in some populations there are haplotypes that lack this locus (Norman et al. 2007), and this appears to be the case for a small subset of individuals in this sample, occurring on one in five haplotypes in the most extreme case; however, the deletion is much rarer or absent in most of the populations examined here (detailed below in the “Results” section). Therefore, we felt that a direct counting approach that necessarily assumed the locus to be present on all haplotypes, while subject to some error, would provide a more accurate estimate of gene frequencies for this locus than simple estimation from carrier frequencies. The frequencies obtained in this manner were in general agreement with those inferred via haplotype estimation in which *KIR*3DL1S1 was estimated to be absent at moderate frequencies (detailed below).

A two-dimensional clustered heatmap for *KIR* carrier frequencies was constructed using the “heatmap” function in the base “stats” package for the R language for statistical computing (R Development Core Team 2008). Briefly, a hierarchical clustering was performed on a set of dissimilarities based on carrier frequencies for the *KIR* loci; both loci and populations were clustered in this manner, and frequency differences were illustrated via the default heatmap color gradient.

We have developed an implementation of the expectation-maximization (EM) algorithm for haplotype frequency estimation that accommodates KIR-specific constraints. Our constrained EM algorithm uses an a priori list of known/possible haplotypes to restrict the space of possible haplotype patterns due to known constraints on gene content variation and allelic variation. The constrained approach differs from traditional EM implementations, in which the set of all possible haplotypic combinations is generated from the observed genotypic data with no restrictions on possible haplotype patterns. The list of user-designated a priori haplotypes is said to “span” the set of observed genotypes in a study if all observed genotypes can be generated from at least one pair of haplotypes in the a priori list. If pairs of haplotypes from the a priori list do not account for all of the observed genotypes in the sample (i.e., the a priori list does not span the observed genotypes), care must be taken in the interpretation of the resulting estimates. Our algorithm uses sharp constraints, meaning that if the a priori haplotypes do not span the observed genotypes a warning/error message is produced and the a priori set must be increased. We began with an a priori list of haplotypes taken from published data based on sequence analysis of seven *KIR* gene content haplotypes (described above; Pyo et al. 2010) and used the program HAPLO-IHP (Yoo et al. 2007) to identify a minimal set of additional haplotypes necessary to span the observed genotypes, based upon the set of “constructed” haplotypes generated by the program. Examination of the extended haplotype output from HAPLO-IHP suggested that one haplotype not included in the initial list may be present at relatively high frequencies in some populations; this haplotype is identical to the extended B haplotype (CenB1 ~ TelB, Fig. 1), with the exception of a deletion of the *KIR*3DS1 locus, and has been previously observed in some populations, as described above (Norman et al. 2007). In order to account for the likelihood that this haplotype is present at appreciable frequencies in some populations in the HGDP-CEPH panel, haplotype frequencies were re-estimated with an amended input set (to include the *KIR*3DS1 deletion haplotype in addition to the seven haplotypes given in Pyo et al. 2010) via our method for the final frequency estimates, with considerably improved resolution in several populations.

Global heatmaps for haplotype frequency data were generated using Generic Mapping tools (Smith and Wessel 1990; Wessel and Smith 1998), an open source collection of tools used to manipulate geographic data sets. *KIR* haplotype frequency data were plotted using an adaptation of a shell script written by Owen Solberg for analysis of *HLA* allele data (www.pypop.org/popdata).

Distance from Africa was calculated using Addis Abbaba, Ethiopia as the central point (latitude 9 N, longitude 38 E) and the latitude and longitude for each population sample (Table 1), computed with the “distance” function in the GenAlEx package (Peakall and Smouse 2006). The carrier frequency of *KIR*3DS1 for each population was plotted against distance from Africa using the “ggplot 2” package for R (Wickham 2009). Data were analyzed for correlations between the frequencies of *KIR*3DS1 and their distance from Africa using the “cor” function in the R base package (Williams and Templeton 2003), as well as plotting and fitting of the regression line. In order to account for the non-independence of the study populations, testing of the statistical significance for the calculated correlation coefficients was accomplished via an empirical approach (Single et al. 2008). Briefly, empirical distributions for the correlation coefficients between *KIR* population frequencies and distances from Africa were generated from 4,132 genome-wide SNP markers from the HGDP-CEPH Genome Diversity Panel Database version 2.0 (ftp://ftp.cephb.fr/hgdp_v2) across all study populations. Only individuals for whom *KIR* genotyping was performed were included in the analysis of the SNP data. Empirical *p* values (*p*_emp_) represent the proportion of the distribution of the correlations for the additional markers that were greater in absolute value than the true correlation.

A similar approach was employed to obtain an empirical distribution for FST values from the same markers. Here, FST was calculated according to Nei (1987) using an in-house R script and *p*_emp_ represents the proportion of the (two-tailed) distribution above or below the FST values obtained for the *KIR* loci.

## Results

### KIR genotypic variation in the HGDP-CEPH

**Fig. 2:**
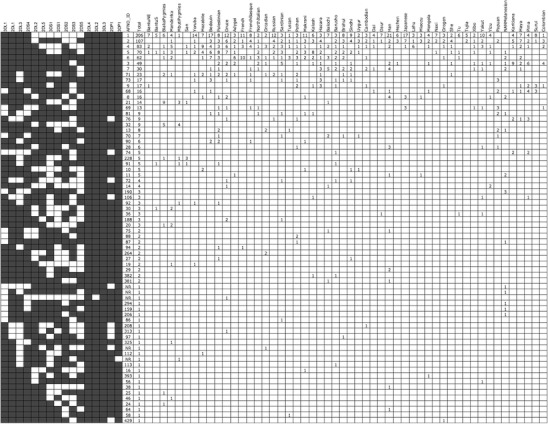
*KIR* genotypes detected and the number of observations in the HGDP-CEPH populations. Loci are arranged in accordance with previously reported genotypes in the allelefrequencies.net database (AFND), and the AFND identification number is given where applicable (AFND_ID)

Each *KIR* gene content profile (genotype) detected and the number of observations in the HGDP-CEPH population are shown in Fig. 2. For comparison purposes, the loci are arranged in accordance with previously reported genotypes in the allelefrequencies.net database (AFND), and the AFND identification number is given where applicable. Seventy unique genotypes are present in the 52 populations under study. Among these 70 genotypic profiles, 29 are observed in only one population; the majority (*n* = 25) is seen in only one individual, and five genotypes have not been previously reported in 108 populations listed on AFND. The six most frequent *KIR* genotypes in the HGDP-CEPH each have a frequency of ≥5 % worldwide, and together account for over two thirds of all genotypes observed in the panel (Table 2). These six genotypes correspond to the previously observed genotypes numbered one through six in the AFND and are the most common genotypes in the database. The most common gene content profile corresponds to homozygosity for the A haplotype (A/A) and is present in all but 3 of the 52 HGDP-CEPH populations. The two Oceanic populations (NAN Melanesian and Papua New Guinea) and one African population (San) did not have any individuals sampled with this profile; in contrast, of 108 populations listed in the AFND for whom *KIR* genotypes were reported in the literature, all report observations of this genotype. Indeed, the San, Papuan, and NAN Melanesian populations had very few individuals with any of the top six genotypes. In the Mbuti Pygmies, only one individual tested positive for the A/A genotype, and this population lacked any other individuals bearing any of the six most common genotypes. Seven of 29 “private” genotypes (observed in only one population) in the diversity panel are observed in the NAN Melanesian sample with one of these found in three individuals. In addition, two of the NAN Melanesian private genotypes have not been previously reported in any population tested to date. Three other previously unreported genotypes were detected in the HGDP-CEPH: one each in the Druze (Middle East), Orcadian (Europe), and Mbuti Pygmies (Africa) populations.

**Table 2:** Frequency of the six most common genotypes worldwide, with associated Cen and Tel haplotype structures

AFND ID^a^	Cen	Tel	AFND populations^b^ (*n*)	HGDP-CEPH populations (*n*)	Worldwide (*f*)	Africa (*f*)	Mideast (*f*)	Europe (*f*)	CSAsia (*f*)	EAsia (*f*)	Oceania (*f*)	America (*f*)
1	A/A	A/A	108	49	0.314	0.327	0.370	0.284	0.278	0.460	0.000	0.240
2	A/A	A/B	97	41	0.110	0.018	0.101	0.101	0.111	0.160	0.054	0.180
4	A/B2	A/A	99	39	0.085	0.088	0.185	0.122	0.039	0.084	0.000	0.060
5	A/B1	A/A	90	28	0.072	0.080	0.210	0.054	0.117	0.025	0.027	0.000
6	A/B1	A/B	86	25	0.064	0.027	0.092	0.176	0.083	0.013	0.054	0.020
3	A/B2	A/B	88	23	0.050	0.000	0.034	0.068	0.028	0.034	0.027	0.210
Total					0.694	0.540	0.992	0.804	0.656	0.776	0.162	0.710

Counts for the number of populations with observations of a given genotype in the AFND and HGDP panel are shown and worldwide and region-specific frequencies

^a^Genotype ID in the allelefrequencies.net database

^b^Out of 108 population entries in AFND panel

**Fig. 3:**
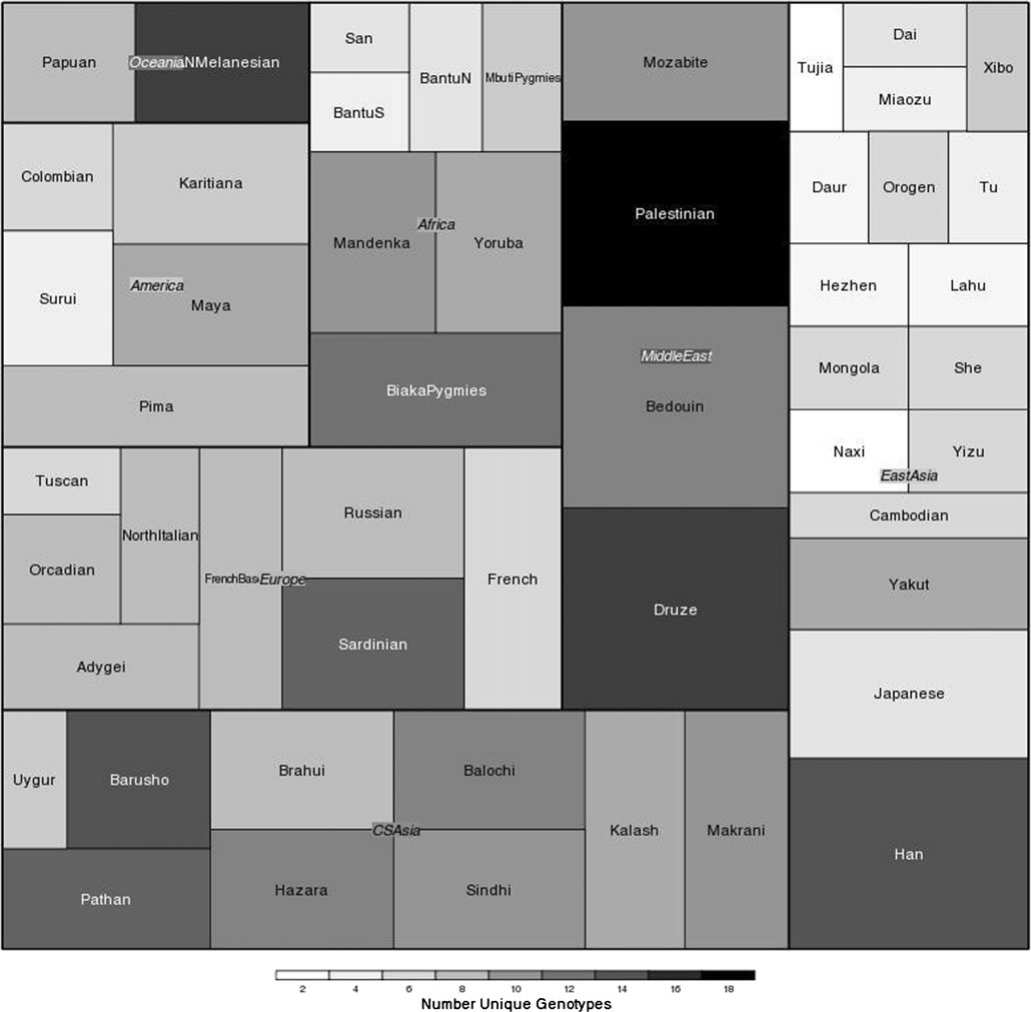
Treemap depicting sample size and genotypic diversity in each HGDP-CEPH population, organized by world region. The *size of the rectangle* for each population or region corresponds to sample size. Variation in the number of genotypes detected in each population is given by grayscale variation

Worldwide, an average of eight unique gene content profiles (genotypes) are observed in each population; however, there is a wide range among populations. Several Asian populations in the sample are extremely homogenous for the *KIR*, with only two or three different genotypes; however, the sample sizes for these populations are among the smallest in the panel. In contrast, the Palestinian population is the most diverse, with 18 unique genotypes, but the sample size is one of the largest in the panel. In order to visualize the relationship between sample size and *KIR* genotypic diversity in the HGDP populations, we constructed a treemap, which displays a graphical representation of hierarchical data via variation in color and area (Fig. 3). While there is a general correlation between populations with larger sample size and greater numbers of KIR genotypes observed, the treemap clearly shows that even within world regions considerable variation exists between populations with similar sample size. This means of visualization also allows ready comparison of sampling variation between world regions. While there is fairly even representation from the major world regions (with the exception of Oceania, with only two populations), it is clear for example that there is an over-representation of East Asian populations, with numerous populations comprised of small numbers of individuals and fewer unique genotypes. In contrast, there are relatively few North African/Middle Eastern populations represented within the diversity panel, but their sample sizes are much larger than most, and many unique genotypes are observed. These observations highlight the need for caution in over-interpretation of the data, particularly when performing analyses between and across world regions, some of which are more or less fully represented in this panel.

### KIR frequency distributions in the HGDP-CEPH

**Fig. 4:**
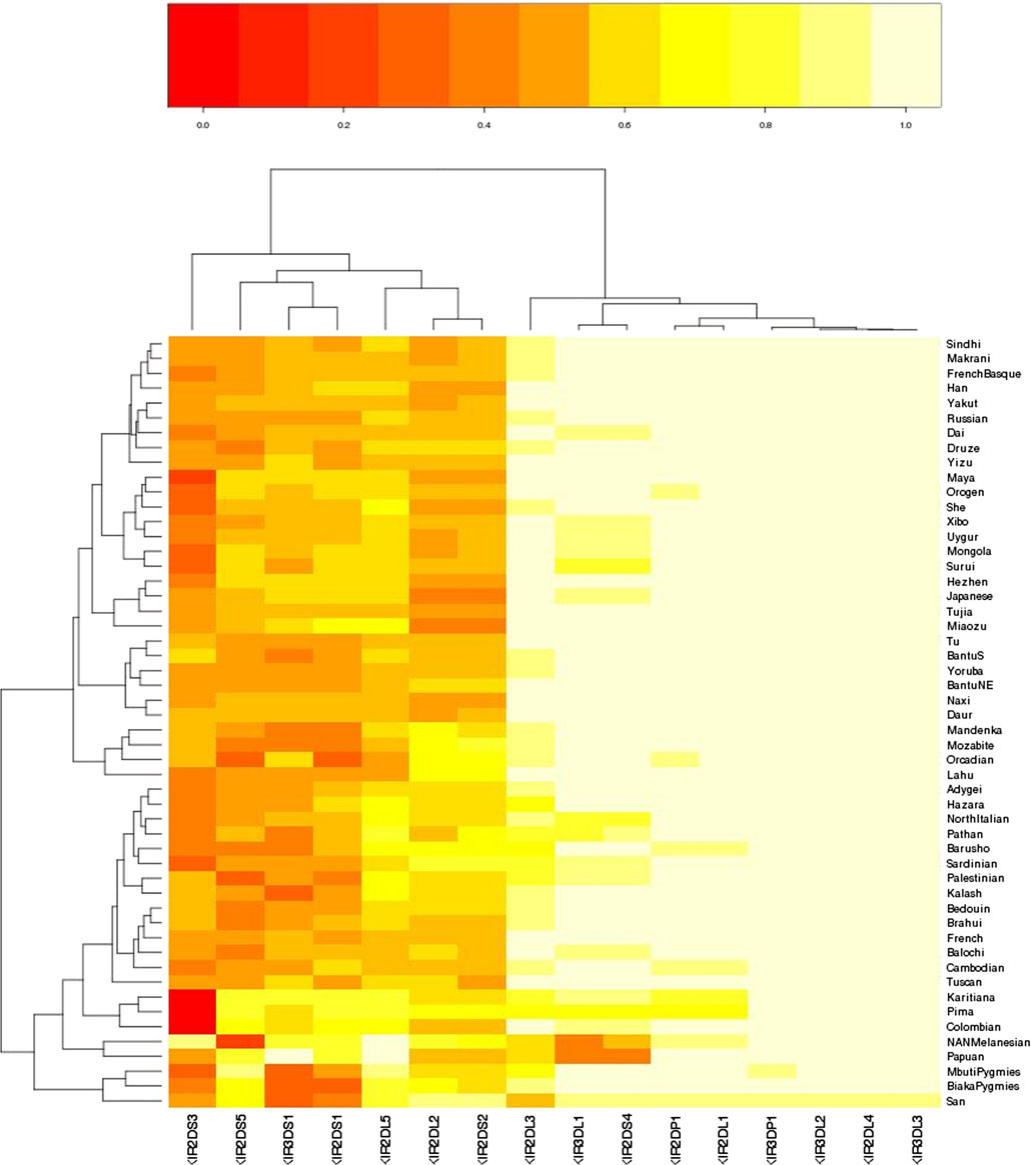
A two-dimensional clustered heatmap based on carrier frequencies for all KIR loci typed in the HGDP-CEPH. KIR loci are clustered along the ***x***-axis and populations along the *y*-axis according to similarities in carrier frequencies of the loci. Variations in carrier frequencies are depicted by the color scale

Gene and carrier frequencies for all of the *KIR* loci typed in the HGDP-CEPH populations are given in Table 3. The two-dimensional clustered heatmap based on carrier frequencies for all KIR loci typed in this study (Fig. 4) allows visualization along the first dimension (*x*-axis, KIR loci) of the strong linkage disequilibrium between the loci associated with the A and B haplotypes that define the two major clades. The heatmap clearly illustrates the much higher frequencies for all A haplotype loci, as well as subnodes within both major clusters along affiliation with the centromeric and telomeric intervals of the KIR region. Clustering in the second dimension (*y*-axis, populations) reveals two major clades according to the relative carrier frequency of the B haplotype loci. Within the larger cluster defined by higher overall carrier frequencies of B-haplotype-associated loci, specific patterns of genotypic variation are clearly seen in the independent clustering of the three African hunter-gather populations (the San and Biaka and Mbuti Pygmies); these three populations are characterized by high overall B haplotype frequencies, with the notable exception of *KIR*2DS1, *KIR*2DS3, and *KIR*3DS1. A separate cluster with the two Oceanic populations is defined by high frequencies for most B haplotype loci. A third cluster is composed of three Amerindian populations (Karitiana, Pima, and Columbian), where *KIR2DS3* is virtually absent. The large cluster associated with generally lower B haplotype frequencies is populated primarily by the remaining African and East Asian populations. The African populations are further distinguished by exceptionally low frequencies of the telomeric B haplotype loci.

**Table 3:** Gene and carrier frequencies for *KIR* loci in the HGDP-CEPH

Population	Frequency	2DL1	2DL2	2DL3	2DL4	2DL5	2DP1	2DS1	2DS2	2DS3	2DS4	2DS5	3DL1	3DL2	3DL3	3DP1	3DS1
Bantu N.E.	Carrier	1.000	0.364	1.000	1.000	0.182	1.000	0.091	0.364	0.091	1.000	0.091	1.000	1.000	1.000	1.000	0.000
	Gene	1.000	0.202	1.000	1.000	0.095	1.000	0.047	0.202	0.047	1.000	0.047	1.000	1.000	1.000	1.000	0.000
Bantu S.	Carrier	1.000	0.250	0.875	1.000	0.375	1.000	0.125	0.250	0.375	1.000	0.125	1.000	1.000	1.000	1.000	0.000
	Gene	1.000	0.134	0.646	1.000	0.209	1.000	0.065	0.134	0.209	1.000	0.065	1.000	1.000	1.000	1.000	0.000
Biaka Pygmies	Carrier	1.000	0.621	0.897	1.000	0.759	1.000	0.034	0.586	0.172	1.000	0.655	1.000	1.000	1.000	1.000	0.069
	Gene	1.000	0.384	0.678	1.000	0.509	1.000	0.017	0.357	0.090	1.000	0.413	1.000	1.000	1.000	1.000	0.035
Mandenka	Carrier	1.000	0.696	0.913	1.000	0.565	1.000	0.217	0.522	0.391	0.957	0.304	1.000	1.000	1.000	1.000	0.174
	Gene	1.000	0.448	0.705	1.000	0.341	1.000	0.115	0.308	0.220	0.791	0.166	1.000	1.000	1.000	1.000	0.091
Mbuti Pygmies	Carrier	1.000	0.583	0.667	1.000	0.917	1.000	0.250	0.583	0.083	1.000	0.917	1.000	1.000	1.000	0.917	0.000
	Gene	1.000	0.355	0.423	1.000	0.711	1.000	0.134	0.355	0.043	1.000	0.711	1.000	1.000	1.000	0.711	0.000
San	Carrier	1.000	1.000	0.429	1.000	0.857	1.000	0.143	1.000	0.286	1.000	0.714	1.000	1.000	1.000	1.000	0.000
	Gene	1.000	1.000	0.244	1.000	0.622	1.000	0.074	1.000	0.155	1.000	0.465	1.000	1.000	1.000	1.000	0.000
Yoruba	Carrier	1.000	0.348	0.870	1.000	0.304	1.000	0.174	0.304	0.174	0.957	0.174	0.957	1.000	1.000	1.000	0.130
	Gene	1.000	0.192	0.639	1.000	0.166	1.000	0.091	0.166	0.091	0.791	0.091	0.791	1.000	1.000	1.000	0.067
Mozabite	Carrier	1.000	0.630	0.889	1.000	0.333	1.000	0.074	0.704	0.333	1.000	0.074	1.000	1.000	1.000	1.000	0.074
	Gene	1.000	0.391	0.667	1.000	0.184	1.000	0.038	0.456	0.184	1.000	0.038	1.000	1.000	1.000	1.000	0.038
Bedouin	Carrier	1.000	0.565	0.848	1.000	0.543	1.000	0.391	0.565	0.457	0.957	0.326	0.957	1.000	1.000	1.000	0.391
	Gene	1.000	0.341	0.610	1.000	0.324	1.000	0.220	0.341	0.263	0.791	0.179	0.791	1.000	1.000	1.000	0.220
Palestinian	Carrier	1.000	0.667	0.810	1.000	0.738	1.000	0.405	0.667	0.571	0.929	0.310	0.929	1.000	1.000	1.000	0.452
	Gene	1.000	0.423	0.564	1.000	0.488	1.000	0.228	0.423	0.345	0.733	0.169	0.733	1.000	1.000	1.000	0.260
Druze	Carrier	0.935	0.500	0.870	1.000	0.478	0.935	0.283	0.500	0.261	0.957	0.239	0.957	0.978	1.000	1.000	0.370
	Gene	0.745	0.293	0.639	1.000	0.278	0.745	0.153	0.293	0.140	0.791	0.128	0.791	0.853	1.000	1.000	0.206
Adygei	Carrier	1.000	0.706	0.941	1.000	0.765	1.000	0.647	0.706	0.529	1.000	0.588	1.000	1.000	1.000	1.000	0.588
	Gene	1.000	0.458	0.757	1.000	0.515	1.000	0.406	0.458	0.314	1.000	0.358	1.000	1.000	1.000	1.000	0.358
French	Carrier	1.000	0.538	0.962	1.000	0.462	1.000	0.423	0.538	0.423	1.000	0.423	1.000	1.000	1.000	1.000	0.462
	Gene	1.000	0.321	0.804	1.000	0.266	1.000	0.240	0.321	0.240	1.000	0.240	1.000	1.000	1.000	1.000	0.266
French Basque	Carrier	1.000	0.409	0.864	1.000	0.455	1.000	0.409	0.409	0.227	0.955	0.318	0.955	1.000	1.000	1.000	0.409
	Gene	1.000	0.231	0.631	1.000	0.261	1.000	0.231	0.231	0.121	0.787	0.174	0.787	1.000	1.000	1.000	0.231
North Italian	Carrier	1.000	0.714	0.929	1.000	0.786	1.000	0.643	0.714	0.500	0.857	0.571	0.857	1.000	1.000	1.000	0.643
	Gene	1.000	0.465	0.733	1.000	0.537	1.000	0.402	0.465	0.293	0.622	0.345	0.622	1.000	1.000	1.000	0.402
Orcadian	Carrier	1.000	0.692	0.846	1.000	0.385	0.923	0.154	0.692	0.462	1.000	0.154	1.000	1.000	1.000	1.000	0.538
	Gene	1.000	0.445	0.608	1.000	0.216	0.723	0.080	0.445	0.266	1.000	0.080	1.000	1.000	1.000	1.000	0.321
Russian	Carrier	0.958	0.333	0.875	1.000	0.417	0.958	0.250	0.333	0.208	1.000	0.250	1.000	1.000	1.000	1.000	0.250
	Gene	0.796	0.184	0.646	1.000	0.236	0.796	0.134	0.184	0.110	1.000	0.134	1.000	1.000	1.000	1.000	0.134
Sardinian	Carrier	0.958	0.792	0.833	1.000	0.667	0.958	0.500	0.792	0.333	0.917	0.500	0.875	1.000	1.000	1.000	0.542
	Gene	0.796	0.544	0.592	1.000	0.423	0.796	0.293	0.544	0.184	0.711	0.293	0.646	1.000	1.000	1.000	0.323
Tuscan	Carrier	1.000	0.750	1.000	1.000	0.750	1.000	0.625	0.625	0.625	1.000	0.625	1.000	1.000	1.000	1.000	0.750
	Gene	1.000	0.500	1.000	1.000	0.500	1.000	0.388	0.388	0.388	1.000	0.388	1.000	1.000	1.000	1.000	0.500
Pathan	Carrier	0.952	0.667	0.810	1.000	0.810	0.952	0.619	0.762	0.476	0.905	0.619	0.857	1.000	1.000	1.000	0.524
	Gene	0.782	0.423	0.564	1.000	0.564	0.782	0.383	0.512	0.276	0.691	0.383	0.622	1.000	1.000	1.000	0.310
Makrani	Carrier	1.000	0.320	0.920	1.000	0.440	1.000	0.360	0.360	0.320	0.960	0.240	0.960	1.000	1.000	1.000	0.360
	Gene	1.000	0.175	0.717	1.000	0.252	1.000	0.200	0.200	0.175	0.800	0.128	0.800	1.000	1.000	1.000	0.200
Kalash	Carrier	0.958	0.625	0.917	1.000	0.750	0.958	0.417	0.625	0.500	0.958	0.417	0.958	1.000	1.000	1.000	0.292
	Gene	0.796	0.388	0.711	1.000	0.500	0.796	0.236	0.388	0.293	0.796	0.236	0.796	1.000	1.000	1.000	0.158
Hazara	Carrier	1.000	0.682	0.818	1.000	0.818	1.000	0.682	0.682	0.455	1.000	0.545	1.000	1.000	1.000	1.000	0.545
	Gene	1.000	0.436	0.574	1.000	0.574	1.000	0.436	0.436	0.261	1.000	0.326	1.000	1.000	1.000	1.000	0.326
Balochi	Carrier	1.000	0.609	1.000	1.000	0.565	1.000	0.522	0.565	0.478	0.870	0.348	0.870	1.000	1.000	1.000	0.565
	Gene	1.000	0.374	1.000	1.000	0.341	1.000	0.308	0.341	0.278	0.639	0.192	0.639	1.000	1.000	1.000	0.341
Barusho	Carrier	0.900	0.750	0.750	1.000	0.750	0.900	0.650	0.750	0.500	0.950	0.500	0.950	1.000	1.000	1.000	0.450
	Gene	0.684	0.500	0.500	1.000	0.500	0.684	0.408	0.500	0.293	0.776	0.293	0.776	1.000	1.000	1.000	0.258
Brahui	Carrier	0.955	0.500	0.864	1.000	0.591	0.955	0.455	0.500	0.455	0.955	0.318	0.955	1.000	1.000	1.000	0.409
	Gene	0.787	0.293	0.631	1.000	0.360	0.787	0.261	0.293	0.261	0.787	0.174	0.787	1.000	1.000	1.000	0.231
Sindhi	Carrier	0.957	0.348	0.870	1.000	0.522	0.957	0.348	0.391	0.304	1.000	0.261	1.000	1.000	1.000	1.000	0.391
	Gene	0.791	0.192	0.639	1.000	0.308	0.791	0.192	0.220	0.166	1.000	0.140	1.000	1.000	1.000	1.000	0.220
Uygur	Carrier	1.000	0.333	1.000	1.000	0.556	1.000	0.444	0.444	0.222	0.889	0.444	0.889	1.000	1.000	1.000	0.444
	Gene	1.000	0.184	1.000	1.000	0.333	1.000	0.255	0.255	0.118	0.667	0.255	0.667	1.000	1.000	1.000	0.255
Cambodian	Carrier	0.909	0.545	0.909	1.000	0.545	0.909	0.636	0.545	0.273	1.000	0.364	1.000	1.000	1.000	1.000	0.455
	Gene	0.698	0.326	0.698	1.000	0.326	0.698	0.397	0.326	0.147	1.000	0.202	1.000	1.000	1.000	1.000	0.261
Dai	Carrier	1.000	0.375	1.000	1.000	0.375	1.000	0.375	0.375	0.125	0.875	0.250	0.875	1.000	1.000	1.000	0.375
	Gene	1.000	0.209	1.000	1.000	0.209	1.000	0.209	0.209	0.065	0.646	0.134	0.646	1.000	1.000	1.000	0.209
Daur	Carrier	1.000	0.000	1.000	1.000	0.222	1.000	0.111	0.111	0.111	1.000	0.111	1.000	1.000	1.000	1.000	0.111
	Gene	1.000	0.000	1.000	1.000	0.118	1.000	0.057	0.057	0.057	1.000	0.057	1.000	1.000	1.000	1.000	0.057
Han	Carrier	1.000	0.326	1.000	1.000	0.500	1.000	0.478	0.261	0.304	0.957	0.217	0.957	1.000	1.000	1.000	0.413
	Gene	1.000	0.179	1.000	1.000	0.293	1.000	0.278	0.140	0.166	0.791	0.115	0.791	1.000	1.000	1.000	0.234
Hezhen	Carrier	1.000	0.100	1.000	1.000	0.400	1.000	0.400	0.100	0.000	1.000	0.400	1.000	1.000	1.000	1.000	0.400
	Gene	1.000	0.051	1.000	1.000	0.225	1.000	0.225	0.051	0.000	1.000	0.225	1.000	1.000	1.000	1.000	0.225
Japanese	Carrier	1.000	0.032	1.000	1.000	0.419	1.000	0.419	0.032	0.097	0.903	0.323	0.903	1.000	1.000	1.000	0.419
	Gene	1.000	0.016	1.000	1.000	0.238	1.000	0.238	0.016	0.050	0.689	0.177	0.689	1.000	1.000	1.000	0.238
Lahu	Carrier	1.000	0.600	1.000	1.000	0.100	1.000	0.100	0.600	0.000	1.000	0.100	1.000	1.000	1.000	1.000	0.100
	Gene	1.000	0.368	1.000	1.000	0.051	1.000	0.051	0.368	0.000	1.000	0.051	1.000	1.000	1.000	1.000	0.051
Miaozu	Carrier	1.000	0.125	1.000	1.000	0.625	1.000	0.625	0.125	0.250	1.000	0.375	1.000	1.000	1.000	1.000	0.500
	Gene	1.000	0.065	1.000	1.000	0.388	1.000	0.388	0.065	0.134	1.000	0.209	1.000	1.000	1.000	1.000	0.293
Mongola	Carrier	1.000	0.300	1.000	1.000	0.500	1.000	0.500	0.400	0.000	0.900	0.500	0.900	1.000	1.000	1.000	0.400
	Gene	1.000	0.163	1.000	1.000	0.293	1.000	0.293	0.225	0.000	0.684	0.293	0.684	1.000	1.000	1.000	0.225
Naxi	Carrier	1.000	0.100	1.000	1.000	0.300	1.000	0.200	0.100	0.100	1.000	0.200	1.000	1.000	1.000	1.000	0.200
	Gene	1.000	0.051	1.000	1.000	0.163	1.000	0.106	0.051	0.051	1.000	0.106	1.000	1.000	1.000	1.000	0.106
Orogen	Carrier	1.000	0.444	1.000	1.000	0.556	0.889	0.556	0.444	0.111	1.000	0.556	1.000	1.000	1.000	1.000	0.444
	Gene	1.000	0.255	1.000	1.000	0.333	0.667	0.333	0.255	0.057	1.000	0.333	1.000	1.000	1.000	1.000	0.255
She	Carrier	1.000	0.400	0.900	1.000	0.700	1.000	0.500	0.400	0.200	1.000	0.500	1.000	1.000	1.000	1.000	0.500
	Gene	1.000	0.225	0.684	1.000	0.452	1.000	0.293	0.225	0.106	1.000	0.293	1.000	1.000	1.000	1.000	0.293
Tu	Carrier	1.000	0.222	1.000	1.000	0.333	1.000	0.111	0.333	0.333	1.000	0.111	1.000	1.000	1.000	1.000	0.111
	Gene	1.000	0.118	1.000	1.000	0.184	1.000	0.057	0.184	0.184	1.000	0.057	1.000	1.000	1.000	1.000	0.057
Tujia	Carrier	1.000	0.000	1.000	1.000	0.286	1.000	0.286	0.000	0.000	1.000	0.286	1.000	1.000	1.000	1.000	0.286
	Gene	1.000	0.000	1.000	1.000	0.155	1.000	0.155	0.000	0.000	1.000	0.155	1.000	1.000	1.000	1.000	0.155
Xibo	Carrier	1.000	0.500	1.000	1.000	0.625	1.000	0.500	0.500	0.250	0.875	0.375	0.875	1.000	1.000	1.000	0.500
	Gene	1.000	0.293	1.000	1.000	0.388	1.000	0.293	0.293	0.134	0.646	0.209	0.646	1.000	1.000	1.000	0.293
Yakut	Carrier	0.955	0.273	0.955	1.000	0.409	0.955	0.364	0.364	0.182	0.955	0.318	0.955	1.000	1.000	1.000	0.318
	Gene	0.787	0.147	0.787	1.000	0.231	0.787	0.202	0.202	0.095	0.787	0.174	0.787	1.000	1.000	1.000	0.174
Yizu	Carrier	1.000	0.300	1.000	1.000	0.300	1.000	0.200	0.300	0.200	1.000	0.100	1.000	1.000	1.000	1.000	0.400
	Gene	1.000	0.163	1.000	1.000	0.163	1.000	0.106	0.163	0.106	1.000	0.051	1.000	1.000	1.000	1.000	0.225
Papuan	Carrier	1.000	0.625	0.688	1.000	1.000	1.000	0.875	0.625	0.563	0.500	0.875	0.500	1.000	1.000	1.000	1.000
	Gene	1.000	0.388	0.441	1.000	1.000	1.000	0.646	0.388	0.339	0.293	0.646	0.293	1.000	1.000	1.000	1.000
NAN Melanesian	Carrier	0.952	0.905	0.714	1.000	1.000	0.952	0.905	0.810	0.952	0.667	0.381	0.524	1.000	1.000	1.000	0.857
	Gene	0.782	0.691	0.465	1.000	1.000	0.782	0.691	0.564	0.782	0.423	0.213	0.310	1.000	1.000	1.000	0.622
Karitiana	Carrier	0.833	0.625	0.833	1.000	0.792	0.833	0.792	0.625	0.000	0.875	0.792	0.875	1.000	1.000	1.000	0.792
	Gene	0.592	0.388	0.592	1.000	0.544	0.592	0.544	0.388	0.000	0.646	0.544	0.646	1.000	1.000	1.000	0.544
Maya	Carrier	0.958	0.417	0.958	1.000	0.625	0.958	0.625	0.417	0.125	0.958	0.583	0.958	1.000	1.000	1.000	0.583
	Gene	0.796	0.236	0.796	1.000	0.388	0.796	0.388	0.236	0.065	0.796	0.355	0.796	1.000	1.000	1.000	0.355
Pima	Carrier	0.760	0.760	0.760	1.000	0.800	0.760	0.800	0.760	0.000	0.760	0.800	0.760	1.000	1.000	1.000	0.680
	Gene	0.510	0.510	0.510	1.000	0.553	0.510	0.553	0.510	0.000	0.510	0.553	0.510	1.000	1.000	1.000	0.434
Surui	Carrier	1.000	0.400	1.000	1.000	0.467	1.000	0.467	0.400	0.000	0.800	0.467	0.800	1.000	1.000	1.000	0.267
	Gene	1.000	0.225	1.000	1.000	0.270	1.000	0.270	0.225	0.000	0.553	0.270	0.553	1.000	1.000	1.000	0.144
Colombian	Carrier	1.000	0.583	1.000	1.000	0.750	1.000	0.750	0.583	0.000	0.917	0.750	0.917	1.000	1.000	1.000	0.667
	Gene	1.000	0.355	1.000	1.000	0.500	1.000	0.500	0.355	0.000	0.711	0.500	0.711	1.000	1.000	1.000	0.423

### KIR haplotypes in the HGDP-CEPH

**Fig. 5:**
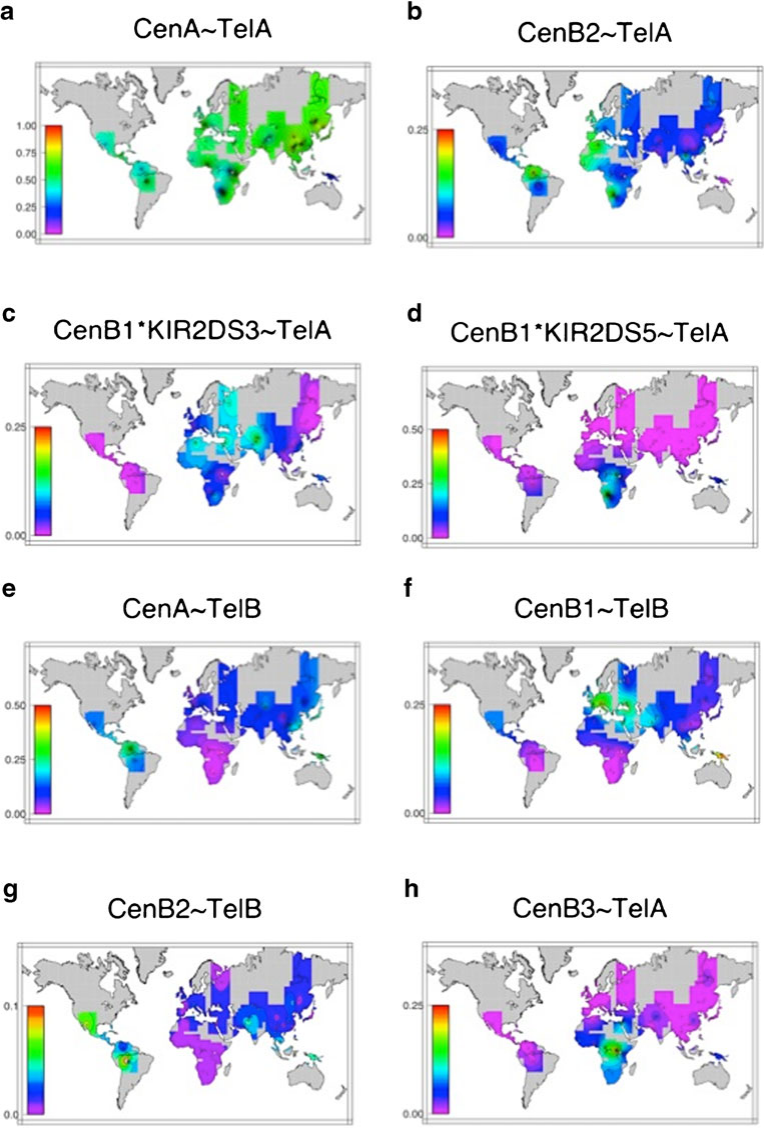
**a**–**h** Worldwide KIR haplotype frequencies, based on population level estimates, in the HGDP-CEPH

Frequency distributions obtained via population level haplotype estimation for the most common haplotypes observed in the HGDP-CEPH (Fig. 1) are shown in Fig. 5a–g. The six gene content haplotypes illustrated in Fig. 1 are all observed at frequencies ≥0.05 in all geographic regions, and combinations of these six correspond to the six most common genotypes worldwide (Fig. 2 and AFND). Together these six haplotypes account for 85 % of the total observed variation in most world regions examined here, with the exception of Africa and Oceania, where extensive diversity in the B haplotype is observed. While haplotype estimation for the more common *KIR* genotypes is relatively robust (Gourraud et al. 2007), considerable phase uncertainty is inherent for the more rare genotypes, due to limited linkage disequilibrium and small sample sizes in many cases; in some populations, up to 15 % of haplotypes are not resolved according to the input list described in “Materials and methods”. While the specifics regarding phase for these haplotypes are unresolved, they are reflected in the less common genotypes shown in Fig. 2.

As noted above, although worldwide approximately 30 % of individuals are homozygous for the canonical A haplotype (*f* = 0.54), a wide range in the A haplotype frequency is observed between populations, from 8 to 80 % (Fig. 5a). The frequency of Cen-A is generally stable in the range 50–60 % in most populations worldwide, with the exception of within East Asia, where Cen-A is observed at frequencies of greater than 80 %; this is evidenced in the very low frequency of *KIR*2DL2 and *KIR*2DS2 in these populations (Table 3). It is interesting to note, however, that the trend toward the Cen-A motif is not observed in Amerindian populations, suggesting that this shift occurred within East Asia subsequent to the differentiation of Amerindians from these populations.

Perhaps most striking, an unusual pattern of linkage disequilibrium within the centromeric B haplotype is observed in some African populations. While in most African and other HGDP-CEPH populations *KIR*2DS3 is associated with the Cen-B1 motif, three populations, the San, Biaka Pygmy, and Mbuti Pygmy populations are observed to have Cen-B1 haplotypes containing the *KIR*2DS5 allele (Fig. 5c, d). This is evidenced by the much higher frequency of *KIR*2DS5 in these three populations relative to other African populations, in whom the frequency of *KIR*2DS5 and the remaining telomeric B haplotype genes (*KIR*3DS1 and *KIR*2DS1) with which it is generally associated are extremely low. Additionally, the less common Cen-B3 motif, which appears to be a fusion between Cen-A and Cen-B1, and is characterized by the presence of *KIR*2DL3, *KIR*2DL5, *KIR*2DS3S5, and *KIR*2DP1, is observed at very high frequencies in the two pygmy populations (Fig. 5h), and in these populations, this haplotype always includes the *KIR*2DS5 allele.

In contrast, *KIR*2DS5 is almost always observed in Amerindian populations regardless of whether the locus is centromeric or telomeric in the *KIR* gene cluster. Examination of the frequency distribution for *KIR*2DS3S5 in Amerindian populations reveals that *KIR*2DS3 is completely absent in all but one population, the Maya; significantly, previous work has suggested Caucasian admixture in this particular population sample (Rosenberg et al. 2002). While Amerindian populations have both the centromeric (Cen-B1) and telomeric (Tel-B) haplotypic motifs that contain this locus, in nearly all cases only the *KIR*2DS5 allele is present.

While the full-length motif Cen-B1 is very common worldwide, the much shorter Cen-B2 appears to be distributed somewhat sporadically across several world regions. The extended haplotype bearing CenB2 ~ TelB is observed primarily outside of Africa, and this motif largely replaces Cen-B1 in some populations outside of Africa; however, there is no clear pattern or gradient associated with this motif. Again, the less common Cen-B3 is primarily limited to African populations, where as expected substantially greater haplotypic diversity is observed relative to other world regions.

**Fig. 6:**
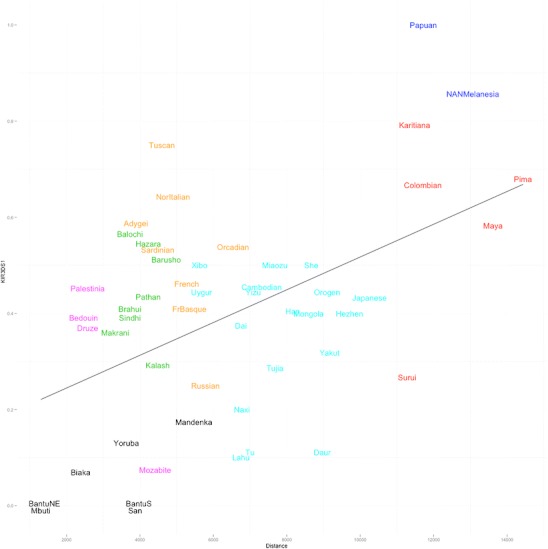
Correlation between *KIR*3DS1 frequencies with distance from Africa. Distance from Africa is plotted along the ***x***-axis and *KIR*3DS1 frequencies along the *y*-axis. A regression line for the data is shown (*r* = 0.49). Populations are colored according to world region: Africa = *black*; Middle East = *magenta*; Europe = *yellow*; Central Asia = *green*; East Asia = *cyan*; Amerindian = *red*; Oceania = *blue*

In contrast to the centromeric *KIR*, the telomeric interval shows a pattern of variation worldwide that generally mirrors human population differentiation. As noted above, two common gene content motifs, Tel-A and Tel-B (Fig. 1), corresponding to the telomeric A and B haplotype, respectively, are observed in most human populations. The telomeric B haplotype loci, marked by *KIR*3DS1, are in strong linkage disequilibrium, with *KIR*3DS1, *KIR*2DL5, *KIR*2DS3S5, and *KIR*2DS1 most often observed as a haplotypic block. While there is a gradient in Tel-B frequencies and a positive correlation (*r* = 0.49) between *KIR*3DS1 carrier frequencies with distance from Africa (Fig. 6), comparison with data from autosomal markers (Conrad et al. 2006) which reflect demographic history, reveals that while this correlation falls on the upper end of the distribution observed for autosomal SNPs, it is not significantly different from the SNP results (*p*_emp_ = 0.20).

### Differentiation of the KIR loci within and between populations and world regions

In general, the centromeric haplotypes are marked by the *KIR*2DL2L3 locus, and the telomeric haplotypes are marked by the *KIR*3DL1S1 locus. As gene frequencies give a better view of genetic differentiation than carrier frequencies, we focused on these loci, assuming that the gene frequencies we obtain via direct counting will in general have less missing data regarding gene content on the second chromosome. While there are clear limitations to this assumption (due to genotypes in some populations with deletion of the *KIR*3DL1S1 locus) as noted in the “Materials and methods” section, we determined that genotypes with this deletion are not common in most populations in our sample. Analysis of *KIR*3DL1S1 carrier status in our sample set reveals that only one individual out of 976 tested in the panel has a negative carrier status for both *KIR*3DL1 and *KIR*3DS1, i.e., is homozygous for the deletion in a previously unreported genotype (Fig. 2). While it is likely that there are individuals in our sample who are heterozygous with the deletion on one chromosome masked by the other, the lack of more than one deletion homozygote suggests a rarity that we felt would not excessively skew our frequency calculations.

Genetic distances calculated from these gene frequencies for *KIR*2DL2L3 and *KIR*3DL1S1 are given in Supplemental Table 1. In general, genetic distances based on both *KIR* loci are greater between continents than those between populations within continents, and distances increase with increasing geographical distance from Africa. Examination of the partitioning of genetic variation for each *KIR* region (Table 4) and comparison with data from other autosomal markers (Conrad et al. 2006) reveal that high levels of *between-population* variation are observed for the centromeric B haplotype locus *KIR*2DL2L3 relative to other autosomal markers *within* world regions. The telomeric B haplotype marker *KIR*3DL1S1, on the other hand, varies more substantially *between regions*. The very minimal differentiation at *KIR*3DL1S1 between populations within major world regions (FST = 0.002) is an order of magnitude lower than that which is observed for autosomal markers genome-wide; comparison with FST value SNPs from the HGDP-CEPH database suggests that this FST value is lower than expectations due to chance alone (*p*_emp_ < 0.05), suggesting a history of balancing selection on the locus.

**Table 4:** FST values for selected centromeric and telomeric KIR loci and autosomal SNPs (Conrad et al. 2006) in the HGDP-CEPH

	KIR2DL2L3	KIR3DL1S1	Autosomal SNPs
Within populations	0.926	0.896	0.890
Between populations	0.024	0.002	0.020
Between regions	0.050	0.102	0.090

## Discussion

*KIR* diversity within populations is maintained by variation in gene content, and *KIR* differentiation between populations can be attributed in large part to frequency variation in common, shared haplotypes; this is evidenced by the fact that in most world regions, six genotypes account for the vast majority of observed variation. Nevertheless, numerous other genotypes are observed to varying degrees, highlighting the great degree of plasticity and potential for variation in gene content within the *KIR* cluster. Moreover, the distribution of genotypes in several populations is characterized by the virtual absence of the more common genotypes, suggesting that they are not requisite at the individual or population level.

Particularly striking is the fact that in three population samples in this panel (the two Oceanic populations NAN Melanesian and Papua New Guinea, and the African San), we did not find any individuals bearing the most common *KIR* genotype worldwide, which corresponds to homozygosity for the canonical A haplotype. The extreme examples of the Oceanic populations and the San are representative of a general trend towards region-specific gene content diversity, coupled with higher overall carrier frequencies of some or all stimulatory *KIR*; these populations occupying the more extreme ends of the spectrum of genetic divergence, specifically the (earliest diverged) African populations and the (most recently diverged) Amerindian and Oceanic populations, generally mirror patterns observed genome-wide (Rosenberg et al. 2002). While geographically and evolutionarily highly divergent, these populations share a history of genetic isolation coupled with small effective population size, suggesting a strong influence of genetic drift in the generation of the less common gene content haplotypes. This is highlighted by the presence of a previously unreported genotype in the Orcadian (Scotland) population; while *KIR* genotypes in European populations generally have common *KIR* types, the Orcadian population is particularly isolated relative to the remainder of the continent. Within Oceania, the six most common *KIR* genotypes account for a mere 16 % of the total observed genotypes, compared to greater than 50 % in all other world regions. The NAN Melanesian population in particular is astonishingly diverse, with 15 unique haplotypes detected in a sample of only 21 individuals. Variation specific to the centromeric and telomeric regions of the *KIR* haplotype in the African and Amerindian populations is responsible for the delineation of discrete clades upon clustering by carrier frequencies, and while the Amerindian populations do not exhibit exceptionally high levels of genotypic diversity, they display distinctive patterns unique to the region. In general, patterns of variation in these highly divergent populations are simply the most extreme examples of more general worldwide trends with regard to *KIR* gene content.

While *KIR* genotypic variation in the HGDP-CEPH closely aligns with that reported in other population studies, analysis of KIR frequencies within the panel reveals novel observations and permits a more detailed understanding of particular region-specific and worldwide patterns. As previously noted, the stimulatory *KIR*3DS1 and *KIR*2DS1 are present at much lower frequencies in African populations (Single et al. 2007a; Hollenbach et al. 2010). As a result, the extensive variation and high frequency of *KIR* B haplotypes observed within Africa is defined by higher frequencies of stimulatory loci within the centromeric *KIR*, and gene content variation within the Cen-B1 haplotype is responsible for several of the less common genotypes observed within Africa.

Of particular note is the presence of *KIR*2DS5 on the Cen-B1 haplotype within Africa. While *KIR*2DS5 is known to be an allele of a duplicated locus observed within both the centromeric and telomeric *KIR* haplotype (Ordonez et al. 2008), within Africa, it is most often observed in association with the centromeric KIR loci; in the remainder of world populations (with the exception of Amerindian populations), the *KIR*2DS3 allele is much more likely to be present on the centromeric haplotype. While previous work has suggested that either allele may have originated on Cen-B1 (Pyo et al. 2010), our data point to an early appearance of *KIR*2DS5 in Africa on this section of the *KIR* haplotype. The San, Biaka Pygmy, and Mbuti Pygmy populations, in whom *KIR*2DS5 is observed at very high frequencies on Cen-B1, are those that have been previously identified as the earliest diverged human populations in the HGDP-CEPH and, due to their long history of genetic isolation, may have maintained a vestigial allelic state on Cen-B1. Several explanations have been postulated for the history of the duplication of this locus in the *KIR* cluster (Kelley et al. 2005; Pyo et al. 2010); however, the appearance of the telomeric B haplotype bearing *KIR*2DS5 in populations primarily outside of Africa suggests duplication of the locus prior to the appearance of *KIR*2DS3. Subsequent replacement of *KIR*2DS5 on Cen-B1 by *KIR*2DS3 suggests a selective advantage either of *KIR*2DS3 specifically in this position or the extended haplotype on which it resides, in most world populations. Further, the very high frequency of Cen-B3 bearing *KIR*2DS5 in the pygmy populations points to an early appearance of this haplotype, and it is possible that this represents a transitional haplotype between Cen-A and Cen-B1.

Outside of Africa, this pattern of linkage disequilibrium placing *KIR*2DS5 within the centromeric B haplotype is most often observed in Amerindian populations, where *KIR*2DS3 is almost entirely absent. In addition, the truncated Cen-B2 haplotype, which lacks entirely the *KIR*2DS3S5 locus, is observed at the highest frequencies worldwide in the Amerindian populations. The fact that *KIR*2DS3 is observed at moderate frequencies in East Asian populations suggests that subsequent to New World migration and divergence of Amerindian populations, a selective event or population bottleneck resulted in the loss of *KIR*2DS3. While the high frequency of Cen-B2 could be explained by either evolutionary event, the fixation of the *KIR*2DS5 allele at the *KIR*2DS3S5 locus, particularly when found within the centromeric region (where most world populations bear *KIR*2DS3), suggests negative selection on *KIR*2DS3 in Amerindians.

Further analysis of frequency distributions for the centromeric *KIR* reveals that in most populations there is a very even balance between the inhibitory A haplotypic structure and the more stimulatory B haplotype, except within East Asia, where Cen-A predominates. These data are suggestive of a selective sweep within the centromeric *KIR* in East Asian populations, resulting in near fixation of the Cen-A motif. Previous work in the HGDP-CEPH and HapMap populations has suggested that recent sweeps of this nature have been relatively common in East Asia, compared to other world populations (Lee et al. 2010). This pattern, in combination with that observed in the Amerindians, as well as overall greater within- and between-population variation and higher heterozygosity for the centromeric loci, implies that the centromeric section of the *KIR* cluster has been repeatedly subjected to purifying selection or population bottlenecks throughout human population differentiation.

While present only at very low frequencies in African populations, the telomeric B haplotype loci are seen at increasing frequencies in populations outside of Africa, with a positive correlation of *KIR*3DS1 frequencies with distance from Africa, as noted previously (Single et al. 2007a, b). These frequencies tend to vary significantly between world regions but very little within regions, as evidenced by very low between-population FST values. Geographic clines like that observed for the Tel-B loci can be observed throughout the genome and may be related to a form of genetic drift, “allele-surfing”, in which a low-frequency allele can ride the “wave” of population expansion at the interface of a recent migratory event to obtain a higher frequency in the secondary population. While comparison with neutral markers in these populations does not give clear evidence for selection, this does not rule out selective processes for these alleles. Population studies with larger sample sizes will help to refine these results. Lee et al. (2010), in analyzing FST values for putatively selected variants in the HGDP-CEPH and HapMap populations at various geographic scales, noted that neutral processes related to population history and human migrations powerfully influence the frequencies of even selected alleles and may mitigate the effects of selection to an extent. Regardless, the trend toward increasing frequency for Tel-B, coupled with strong linkage disequilibrium and extremely low FST values, suggests ongoing balancing selection on the telomeric *KIR*, in keeping with previous data for sequence level variation in *KIR*3DL1S1 (Norman et al. 2007). In addition, our analysis of *KIR* genotypes and other previous work (Middleton et al. 2007b) has shown that the region of the telomeric *KIR* including *KIR*3DL1S1 appears to be particularly prone to deletion (and likely duplication) events, contributing substantially to gene content variation. In the HGDP-CEPH populations outside of Africa, most notably the Oceanic and Amerindian populations, we observed extensive variation in gene content in the telomeric *KIR*, further supporting the notion of balancing, or diversifying selection on this section of the *KIR*.

In conclusion, analysis of our data for the *KIR* in the HGDP-CEPH reveals significant evidence for balancing, diversifying selection within the telomeric region, and purifying selection and/or a history of population bottlenecks within the centromeric region, in general accordance with recent work showing different evolutionary histories for the centromeric and telomeric *KIR* (Pyo et al. 2010). In every major world region, while the majority of *KIR* variation in many populations can be attributed to six common gene content profiles, numerous other genotypes are observed worldwide to varying degrees, with the most isolated and diverged populations exhibiting the most divergent *KIR* profiles. Although the ubiquity of the common *KIR* genotypes, and in particular the *KIR* A haplotype, suggests selection for these common types, the fact that we observed three populations lacking A/A homozygous individuals suggests that a high frequency of the A haplotypes is not necessarily requisite in a population. The most extreme variation in B haplotype gene content in populations likely to have been subject to strong genetic drift suggests recent and frequent recombination, with duplication and deletion events magnified by that drift. However, extensive variation was observed in populations worldwide, with previously unreported genotypes observed in populations from four of the seven world regions examined, emphasizing the continuing potential for diversification in the *KIR* region in human populations.

## Electronic supplementary material

Below is the link to the electronic supplementary material.

ESM 1 (DOC 288 kb)
