# Supplementary material for: Killer cell immunoglobulin-like receptor (KIR) gene content variation in the HGDP-CEPH populations
Source: Immunogenetics. 2012 Jul 1;64(10):719–37. doi: 10.1007/s00251-012-0629-x (PMC3438391; doi:10.1007/s00251-012-0629-x)
Supplement: Supplementary file 1 — (DOC 288 kb) [file 251_2012_629_MOESM1_ESM.doc]

Table 1 Supplemental. Genetic distances claculated from gene frequencies for KIR2DL2L3 and KIR3DL1S1 in the CEPH-HGDP.

|  | Bantu N.E. | Bantu S | Biaka Pygmies | Mandenka | Mbuti Pygmies | San | Yoruba | Mozabite | Bedouin | Palestinian | Druze | Adygei | French | French Basque | North Italian | Orcadian | Russian | Sardinian | Tuscan | Pathan | Makrani | Kalash | Hazara | Balochi | Barusho | Brahui | Sindhi | Uygur | Cambodian | Dai | Daur | Han | Hezhen | Japanese | Lahu | Miaozu | Mongola | Naxi | Orogen | She | Tu | Tujia | Xibo | Yakut | Yizu | Papuan | NAN Melanesian | Karitiana | Maya | Pima | Surui | Colombian |
| --- | --- | --- | --- | --- | --- | --- | --- | --- | --- | --- | --- | --- | --- | --- | --- | --- | --- | --- | --- | --- | --- | --- | --- | --- | --- | --- | --- | --- | --- | --- | --- | --- | --- | --- | --- | --- | --- | --- | --- | --- | --- | --- | --- | --- | --- | --- | --- | --- | --- | --- | --- | --- |
| Bantu N.E. | 0.000 |  |  |  |  |  |  |  |  |  |  |  |  |  |  |  |  |  |  |  |  |  |  |  |  |  |  |  |  |  |  |  |  |  |  |  |  |  |  |  |  |  |  |  |  |  |  |  |  |  |  |  |
| Bantu S | 0.000 | 0.000 |  |  |  |  |  |  |  |  |  |  |  |  |  |  |  |  |  |  |  |  |  |  |  |  |  |  |  |  |  |  |  |  |  |  |  |  |  |  |  |  |  |  |  |  |  |  |  |  |  |  |
| Biaka Pygmies | 0.031 | 0.028 | 0.000 |  |  |  |  |  |  |  |  |  |  |  |  |  |  |  |  |  |  |  |  |  |  |  |  |  |  |  |  |  |  |  |  |  |  |  |  |  |  |  |  |  |  |  |  |  |  |  |  |  |
| Mandenka | 0.008 | 0.007 | 0.017 | 0.000 |  |  |  |  |  |  |  |  |  |  |  |  |  |  |  |  |  |  |  |  |  |  |  |  |  |  |  |  |  |  |  |  |  |  |  |  |  |  |  |  |  |  |  |  |  |  |  |  |
| Mbuti Pygmies | 0.070 | 0.064 | 0.012 | 0.042 | 0.000 |  |  |  |  |  |  |  |  |  |  |  |  |  |  |  |  |  |  |  |  |  |  |  |  |  |  |  |  |  |  |  |  |  |  |  |  |  |  |  |  |  |  |  |  |  |  |  |
| San | 0.038 | 0.034 | 0.002 | 0.020 | 0.006 | 0.000 |  |  |  |  |  |  |  |  |  |  |  |  |  |  |  |  |  |  |  |  |  |  |  |  |  |  |  |  |  |  |  |  |  |  |  |  |  |  |  |  |  |  |  |  |  |  |
| Yoruba | 0.003 | 0.002 | 0.026 | 0.002 | 0.059 | 0.031 | 0.000 |  |  |  |  |  |  |  |  |  |  |  |  |  |  |  |  |  |  |  |  |  |  |  |  |  |  |  |  |  |  |  |  |  |  |  |  |  |  |  |  |  |  |  |  |  |
| Mozabite | 0.001 | 0.001 | 0.033 | 0.008 | 0.075 | 0.041 | 0.002 | 0.000 |  |  |  |  |  |  |  |  |  |  |  |  |  |  |  |  |  |  |  |  |  |  |  |  |  |  |  |  |  |  |  |  |  |  |  |  |  |  |  |  |  |  |  |  |
| Bedouin | 0.029 | 0.026 | 0.034 | 0.008 | 0.054 | 0.037 | 0.014 | 0.026 | 0.000 |  |  |  |  |  |  |  |  |  |  |  |  |  |  |  |  |  |  |  |  |  |  |  |  |  |  |  |  |  |  |  |  |  |  |  |  |  |  |  |  |  |  |  |
| Palestinian | 0.035 | 0.032 | 0.042 | 0.012 | 0.063 | 0.045 | 0.018 | 0.030 | 0.001 | 0.000 |  |  |  |  |  |  |  |  |  |  |  |  |  |  |  |  |  |  |  |  |  |  |  |  |  |  |  |  |  |  |  |  |  |  |  |  |  |  |  |  |  |  |
| Druze | 0.019 | 0.017 | 0.034 | 0.006 | 0.064 | 0.040 | 0.008 | 0.015 | 0.003 | 0.004 | 0.000 |  |  |  |  |  |  |  |  |  |  |  |  |  |  |  |  |  |  |  |  |  |  |  |  |  |  |  |  |  |  |  |  |  |  |  |  |  |  |  |  |  |
| Adygei | 0.092 | 0.085 | 0.067 | 0.046 | 0.063 | 0.063 | 0.064 | 0.088 | 0.019 | 0.018 | 0.034 | 0.000 |  |  |  |  |  |  |  |  |  |  |  |  |  |  |  |  |  |  |  |  |  |  |  |  |  |  |  |  |  |  |  |  |  |  |  |  |  |  |  |  |
| French | 0.042 | 0.038 | 0.036 | 0.014 | 0.050 | 0.038 | 0.024 | 0.039 | 0.002 | 0.002 | 0.008 | 0.010 | 0.000 |  |  |  |  |  |  |  |  |  |  |  |  |  |  |  |  |  |  |  |  |  |  |  |  |  |  |  |  |  |  |  |  |  |  |  |  |  |  |  |
| French Basque | 0.031 | 0.028 | 0.037 | 0.010 | 0.058 | 0.040 | 0.015 | 0.028 | 0.000 | 0.000 | 0.003 | 0.018 | 0.002 | 0.000 |  |  |  |  |  |  |  |  |  |  |  |  |  |  |  |  |  |  |  |  |  |  |  |  |  |  |  |  |  |  |  |  |  |  |  |  |  |  |
| North Italian | 0.102 | 0.096 | 0.078 | 0.054 | 0.077 | 0.075 | 0.070 | 0.097 | 0.022 | 0.019 | 0.038 | 0.004 | 0.015 | 0.021 | 0.000 |  |  |  |  |  |  |  |  |  |  |  |  |  |  |  |  |  |  |  |  |  |  |  |  |  |  |  |  |  |  |  |  |  |  |  |  |  |
| Orcadian | 0.029 | 0.029 | 0.050 | 0.017 | 0.093 | 0.062 | 0.018 | 0.023 | 0.012 | 0.011 | 0.006 | 0.046 | 0.016 | 0.012 | 0.050 | 0.000 |  |  |  |  |  |  |  |  |  |  |  |  |  |  |  |  |  |  |  |  |  |  |  |  |  |  |  |  |  |  |  |  |  |  |  |  |
| Russian | 0.010 | 0.009 | 0.024 | 0.002 | 0.053 | 0.029 | 0.003 | 0.009 | 0.005 | 0.008 | 0.002 | 0.041 | 0.011 | 0.006 | 0.049 | 0.011 | 0.000 |  |  |  |  |  |  |  |  |  |  |  |  |  |  |  |  |  |  |  |  |  |  |  |  |  |  |  |  |  |  |  |  |  |  |  |
| Sardinian | 0.065 | 0.060 | 0.050 | 0.028 | 0.058 | 0.050 | 0.041 | 0.061 | 0.008 | 0.006 | 0.017 | 0.006 | 0.004 | 0.007 | 0.005 | 0.028 | 0.024 | 0.000 |  |  |  |  |  |  |  |  |  |  |  |  |  |  |  |  |  |  |  |  |  |  |  |  |  |  |  |  |  |  |  |  |  |  |
| Tuscan | 0.117 | 0.110 | 0.084 | 0.064 | 0.082 | 0.083 | 0.084 | 0.110 | 0.029 | 0.026 | 0.045 | 0.003 | 0.017 | 0.028 | 0.005 | 0.052 | 0.057 | 0.010 | 0.000 |  |  |  |  |  |  |  |  |  |  |  |  |  |  |  |  |  |  |  |  |  |  |  |  |  |  |  |  |  |  |  |  |  |
| Pathan | 0.089 | 0.082 | 0.060 | 0.043 | 0.055 | 0.055 | 0.060 | 0.085 | 0.019 | 0.017 | 0.033 | 0.004 | 0.012 | 0.018 | 0.003 | 0.050 | 0.040 | 0.003 | 0.008 | 0.000 |  |  |  |  |  |  |  |  |  |  |  |  |  |  |  |  |  |  |  |  |  |  |  |  |  |  |  |  |  |  |  |  |
| Makrani | 0.021 | 0.019 | 0.037 | 0.006 | 0.063 | 0.041 | 0.009 | 0.018 | 0.001 | 0.002 | 0.002 | 0.027 | 0.005 | 0.001 | 0.031 | 0.009 | 0.003 | 0.014 | 0.040 | 0.027 | 0.000 |  |  |  |  |  |  |  |  |  |  |  |  |  |  |  |  |  |  |  |  |  |  |  |  |  |  |  |  |  |  |  |
| Kalash | 0.029 | 0.025 | 0.025 | 0.007 | 0.038 | 0.025 | 0.015 | 0.027 | 0.002 | 0.005 | 0.006 | 0.019 | 0.004 | 0.003 | 0.024 | 0.021 | 0.006 | 0.009 | 0.032 | 0.016 | 0.004 | 0.000 |  |  |  |  |  |  |  |  |  |  |  |  |  |  |  |  |  |  |  |  |  |  |  |  |  |  |  |  |  |  |
| Hazara | 0.087 | 0.080 | 0.068 | 0.043 | 0.065 | 0.063 | 0.060 | 0.083 | 0.017 | 0.016 | 0.032 | 0.001 | 0.009 | 0.016 | 0.005 | 0.046 | 0.039 | 0.006 | 0.005 | 0.004 | 0.025 | 0.017 | 0.000 |  |  |  |  |  |  |  |  |  |  |  |  |  |  |  |  |  |  |  |  |  |  |  |  |  |  |  |  |  |
| Balochi | 0.060 | 0.056 | 0.063 | 0.028 | 0.079 | 0.065 | 0.037 | 0.055 | 0.007 | 0.004 | 0.014 | 0.011 | 0.006 | 0.006 | 0.008 | 0.022 | 0.023 | 0.003 | 0.016 | 0.010 | 0.010 | 0.012 | 0.010 | 0.000 |  |  |  |  |  |  |  |  |  |  |  |  |  |  |  |  |  |  |  |  |  |  |  |  |  |  |  |  |
| Barusho | 0.071 | 0.065 | 0.059 | 0.034 | 0.058 | 0.054 | 0.048 | 0.069 | 0.013 | 0.013 | 0.023 | 0.005 | 0.009 | 0.012 | 0.009 | 0.041 | 0.029 | 0.005 | 0.013 | 0.004 | 0.019 | 0.010 | 0.004 | 0.010 | 0.000 |  |  |  |  |  |  |  |  |  |  |  |  |  |  |  |  |  |  |  |  |  |  |  |  |  |  |  |
| Brahui | 0.034 | 0.031 | 0.042 | 0.012 | 0.060 | 0.043 | 0.018 | 0.031 | 0.001 | 0.001 | 0.004 | 0.016 | 0.002 | 0.001 | 0.019 | 0.014 | 0.008 | 0.007 | 0.026 | 0.016 | 0.002 | 0.003 | 0.014 | 0.005 | 0.009 | 0.000 |  |  |  |  |  |  |  |  |  |  |  |  |  |  |  |  |  |  |  |  |  |  |  |  |  |  |
| Sindhi | 0.024 | 0.021 | 0.036 | 0.007 | 0.062 | 0.041 | 0.011 | 0.020 | 0.001 | 0.002 | 0.001 | 0.025 | 0.004 | 0.002 | 0.032 | 0.008 | 0.003 | 0.013 | 0.036 | 0.027 | 0.001 | 0.004 | 0.023 | 0.011 | 0.017 | 0.002 | 0.000 |  |  |  |  |  |  |  |  |  |  |  |  |  |  |  |  |  |  |  |  |  |  |  |  |  |
| Uygur | 0.046 | 0.042 | 0.038 | 0.016 | 0.050 | 0.038 | 0.026 | 0.042 | 0.003 | 0.002 | 0.010 | 0.011 | 0.003 | 0.003 | 0.011 | 0.022 | 0.015 | 0.002 | 0.019 | 0.008 | 0.007 | 0.004 | 0.011 | 0.003 | 0.008 | 0.003 | 0.008 | 0.000 |  |  |  |  |  |  |  |  |  |  |  |  |  |  |  |  |  |  |  |  |  |  |  |  |
| Cambodian | 0.059 | 0.054 | 0.063 | 0.029 | 0.071 | 0.060 | 0.039 | 0.056 | 0.009 | 0.009 | 0.017 | 0.009 | 0.007 | 0.008 | 0.015 | 0.031 | 0.022 | 0.008 | 0.018 | 0.011 | 0.013 | 0.009 | 0.006 | 0.008 | 0.003 | 0.005 | 0.011 | 0.009 | 0.000 |  |  |  |  |  |  |  |  |  |  |  |  |  |  |  |  |  |  |  |  |  |  |  |
| Dai | 0.026 | 0.023 | 0.041 | 0.009 | 0.066 | 0.045 | 0.012 | 0.023 | 0.002 | 0.002 | 0.003 | 0.028 | 0.007 | 0.002 | 0.027 | 0.012 | 0.007 | 0.012 | 0.040 | 0.025 | 0.001 | 0.006 | 0.026 | 0.007 | 0.019 | 0.003 | 0.004 | 0.005 | 0.015 | 0.000 |  |  |  |  |  |  |  |  |  |  |  |  |  |  |  |  |  |  |  |  |  |  |
| Daur | 0.001 | 0.001 | 0.030 | 0.005 | 0.069 | 0.037 | 0.001 | 0.000 | 0.020 | 0.024 | 0.011 | 0.076 | 0.031 | 0.022 | 0.085 | 0.019 | 0.006 | 0.052 | 0.097 | 0.074 | 0.014 | 0.022 | 0.072 | 0.047 | 0.059 | 0.025 | 0.015 | 0.035 | 0.048 | 0.018 | 0.000 |  |  |  |  |  |  |  |  |  |  |  |  |  |  |  |  |  |  |  |  |  |
| Han | 0.033 | 0.030 | 0.052 | 0.014 | 0.075 | 0.054 | 0.018 | 0.029 | 0.002 | 0.002 | 0.005 | 0.021 | 0.005 | 0.002 | 0.024 | 0.014 | 0.009 | 0.011 | 0.032 | 0.023 | 0.002 | 0.007 | 0.018 | 0.006 | 0.014 | 0.001 | 0.003 | 0.007 | 0.007 | 0.003 | 0.023 | 0.000 |  |  |  |  |  |  |  |  |  |  |  |  |  |  |  |  |  |  |  |  |
| Hezhen | 0.034 | 0.030 | 0.031 | 0.010 | 0.046 | 0.032 | 0.018 | 0.031 | 0.001 | 0.002 | 0.006 | 0.014 | 0.001 | 0.001 | 0.020 | 0.015 | 0.007 | 0.007 | 0.024 | 0.015 | 0.003 | 0.002 | 0.013 | 0.008 | 0.010 | 0.002 | 0.003 | 0.003 | 0.008 | 0.006 | 0.024 | 0.005 | 0.000 |  |  |  |  |  |  |  |  |  |  |  |  |  |  |  |  |  |  |  |
| Japanese | 0.034 | 0.031 | 0.040 | 0.011 | 0.060 | 0.043 | 0.017 | 0.030 | 0.001 | 0.000 | 0.005 | 0.018 | 0.003 | 0.001 | 0.018 | 0.014 | 0.009 | 0.006 | 0.028 | 0.016 | 0.002 | 0.004 | 0.016 | 0.004 | 0.012 | 0.001 | 0.003 | 0.002 | 0.009 | 0.001 | 0.024 | 0.002 | 0.003 | 0.000 |  |  |  |  |  |  |  |  |  |  |  |  |  |  |  |  |  |  |
| Lahu | 0.001 | 0.001 | 0.031 | 0.006 | 0.070 | 0.038 | 0.001 | 0.000 | 0.022 | 0.026 | 0.012 | 0.080 | 0.033 | 0.023 | 0.089 | 0.020 | 0.006 | 0.054 | 0.101 | 0.078 | 0.015 | 0.023 | 0.075 | 0.049 | 0.062 | 0.027 | 0.017 | 0.037 | 0.050 | 0.019 | 0.000 | 0.025 | 0.026 | 0.026 | 0.000 |  |  |  |  |  |  |  |  |  |  |  |  |  |  |  |  |  |
| Miaozu | 0.061 | 0.056 | 0.063 | 0.029 | 0.071 | 0.061 | 0.040 | 0.058 | 0.008 | 0.007 | 0.018 | 0.006 | 0.005 | 0.007 | 0.010 | 0.029 | 0.023 | 0.006 | 0.013 | 0.010 | 0.012 | 0.010 | 0.004 | 0.005 | 0.004 | 0.005 | 0.011 | 0.007 | 0.002 | 0.013 | 0.049 | 0.006 | 0.006 | 0.007 | 0.051 | 0.000 |  |  |  |  |  |  |  |  |  |  |  |  |  |  |  |  |
| Mongola | 0.050 | 0.045 | 0.037 | 0.019 | 0.043 | 0.035 | 0.030 | 0.048 | 0.005 | 0.005 | 0.014 | 0.009 | 0.004 | 0.005 | 0.010 | 0.030 | 0.018 | 0.003 | 0.018 | 0.005 | 0.010 | 0.004 | 0.008 | 0.006 | 0.005 | 0.005 | 0.011 | 0.001 | 0.008 | 0.009 | 0.040 | 0.009 | 0.004 | 0.004 | 0.043 | 0.006 | 0.000 |  |  |  |  |  |  |  |  |  |  |  |  |  |  |  |
| Naxi | 0.006 | 0.005 | 0.025 | 0.001 | 0.057 | 0.031 | 0.001 | 0.004 | 0.009 | 0.013 | 0.004 | 0.052 | 0.017 | 0.010 | 0.060 | 0.013 | 0.001 | 0.033 | 0.070 | 0.051 | 0.005 | 0.011 | 0.049 | 0.030 | 0.039 | 0.013 | 0.006 | 0.020 | 0.031 | 0.009 | 0.002 | 0.013 | 0.012 | 0.013 | 0.003 | 0.031 | 0.024 | 0.000 |  |  |  |  |  |  |  |  |  |  |  |  |  |  |
| Orogen | 0.064 | 0.058 | 0.044 | 0.028 | 0.045 | 0.041 | 0.042 | 0.062 | 0.011 | 0.011 | 0.021 | 0.004 | 0.005 | 0.010 | 0.011 | 0.035 | 0.024 | 0.005 | 0.012 | 0.005 | 0.017 | 0.008 | 0.004 | 0.011 | 0.003 | 0.008 | 0.015 | 0.007 | 0.006 | 0.019 | 0.052 | 0.015 | 0.007 | 0.011 | 0.055 | 0.006 | 0.004 | 0.033 | 0.000 |  |  |  |  |  |  |  |  |  |  |  |  |  |
| She | 0.058 | 0.053 | 0.043 | 0.023 | 0.050 | 0.043 | 0.036 | 0.054 | 0.006 | 0.006 | 0.015 | 0.004 | 0.001 | 0.006 | 0.009 | 0.026 | 0.020 | 0.003 | 0.010 | 0.006 | 0.012 | 0.007 | 0.004 | 0.006 | 0.005 | 0.005 | 0.010 | 0.003 | 0.006 | 0.013 | 0.046 | 0.010 | 0.003 | 0.007 | 0.048 | 0.003 | 0.003 | 0.028 | 0.002 | 0.000 |  |  |  |  |  |  |  |  |  |  |  |  |
| Tu | 0.001 | 0.001 | 0.030 | 0.005 | 0.069 | 0.037 | 0.001 | 0.000 | 0.020 | 0.024 | 0.011 | 0.076 | 0.031 | 0.022 | 0.085 | 0.019 | 0.006 | 0.052 | 0.097 | 0.074 | 0.014 | 0.022 | 0.072 | 0.047 | 0.059 | 0.025 | 0.015 | 0.035 | 0.048 | 0.018 | 0.000 | 0.023 | 0.024 | 0.024 | 0.000 | 0.049 | 0.040 | 0.002 | 0.052 | 0.046 | 0.000 |  |  |  |  |  |  |  |  |  |  |  |
| Tujia | 0.015 | 0.012 | 0.024 | 0.002 | 0.049 | 0.028 | 0.005 | 0.012 | 0.003 | 0.005 | 0.002 | 0.033 | 0.007 | 0.004 | 0.040 | 0.011 | 0.001 | 0.019 | 0.047 | 0.033 | 0.002 | 0.004 | 0.031 | 0.018 | 0.024 | 0.005 | 0.002 | 0.010 | 0.018 | 0.005 | 0.009 | 0.006 | 0.004 | 0.005 | 0.010 | 0.018 | 0.013 | 0.002 | 0.019 | 0.014 | 0.009 | 0.000 |  |  |  |  |  |  |  |  |  |  |
| Xibo | 0.052 | 0.048 | 0.052 | 0.022 | 0.067 | 0.053 | 0.030 | 0.048 | 0.004 | 0.002 | 0.011 | 0.011 | 0.004 | 0.003 | 0.009 | 0.021 | 0.018 | 0.002 | 0.018 | 0.009 | 0.007 | 0.008 | 0.010 | 0.001 | 0.008 | 0.003 | 0.009 | 0.001 | 0.007 | 0.005 | 0.040 | 0.005 | 0.005 | 0.002 | 0.042 | 0.005 | 0.003 | 0.024 | 0.009 | 0.005 | 0.040 | 0.013 | 0.000 |  |  |  |  |  |  |  |  |  |
| Yakut | 0.022 | 0.019 | 0.029 | 0.005 | 0.050 | 0.031 | 0.009 | 0.019 | 0.001 | 0.003 | 0.002 | 0.025 | 0.005 | 0.002 | 0.030 | 0.013 | 0.003 | 0.012 | 0.038 | 0.023 | 0.001 | 0.001 | 0.023 | 0.011 | 0.015 | 0.002 | 0.001 | 0.006 | 0.011 | 0.003 | 0.015 | 0.004 | 0.002 | 0.002 | 0.016 | 0.012 | 0.007 | 0.006 | 0.013 | 0.010 | 0.015 | 0.001 | 0.008 | 0.000 |  |  |  |  |  |  |  |  |
| Yizu | 0.016 | 0.016 | 0.045 | 0.010 | 0.086 | 0.054 | 0.008 | 0.012 | 0.009 | 0.010 | 0.003 | 0.049 | 0.016 | 0.009 | 0.055 | 0.003 | 0.005 | 0.030 | 0.060 | 0.052 | 0.005 | 0.017 | 0.047 | 0.023 | 0.040 | 0.012 | 0.005 | 0.021 | 0.029 | 0.008 | 0.009 | 0.009 | 0.013 | 0.011 | 0.010 | 0.027 | 0.028 | 0.005 | 0.036 | 0.027 | 0.009 | 0.005 | 0.021 | 0.009 | 0.000 |  |  |  |  |  |  |  |
| Papuan | 0.313 | 0.301 | 0.239 | 0.217 | 0.211 | 0.231 | 0.248 | 0.303 | 0.150 | 0.137 | 0.183 | 0.088 | 0.134 | 0.146 | 0.058 | 0.200 | 0.213 | 0.091 | 0.074 | 0.073 | 0.171 | 0.154 | 0.093 | 0.097 | 0.104 | 0.143 | 0.175 | 0.111 | 0.128 | 0.150 | 0.282 | 0.153 | 0.148 | 0.133 | 0.288 | 0.118 | 0.108 | 0.235 | 0.115 | 0.115 | 0.282 | 0.194 | 0.104 | 0.167 | 0.218 | 0.000 |  |  |  |  |  |  |
| NAN Melanesian | 0.202 | 0.194 | 0.203 | 0.142 | 0.204 | 0.200 | 0.155 | 0.192 | 0.086 | 0.074 | 0.105 | 0.058 | 0.082 | 0.082 | 0.034 | 0.118 | 0.131 | 0.053 | 0.053 | 0.048 | 0.095 | 0.095 | 0.057 | 0.045 | 0.059 | 0.077 | 0.099 | 0.065 | 0.065 | 0.080 | 0.178 | 0.076 | 0.091 | 0.072 | 0.182 | 0.060 | 0.067 | 0.146 | 0.075 | 0.072 | 0.178 | 0.119 | 0.053 | 0.098 | 0.125 | 0.032 | 0.000 |  |  |  |  |  |
| Karitiana | 0.186 | 0.176 | 0.130 | 0.114 | 0.110 | 0.123 | 0.142 | 0.179 | 0.068 | 0.063 | 0.088 | 0.020 | 0.051 | 0.065 | 0.017 | 0.105 | 0.105 | 0.030 | 0.015 | 0.018 | 0.083 | 0.065 | 0.024 | 0.042 | 0.027 | 0.059 | 0.078 | 0.047 | 0.041 | 0.079 | 0.162 | 0.071 | 0.060 | 0.063 | 0.167 | 0.041 | 0.043 | 0.126 | 0.032 | 0.037 | 0.162 | 0.095 | 0.045 | 0.076 | 0.118 | 0.043 | 0.043 | 0.000 |  |  |  |  |
| Maya | 0.089 | 0.083 | 0.064 | 0.044 | 0.062 | 0.061 | 0.061 | 0.085 | 0.017 | 0.016 | 0.031 | 0.001 | 0.009 | 0.016 | 0.003 | 0.044 | 0.039 | 0.004 | 0.004 | 0.002 | 0.026 | 0.017 | 0.001 | 0.009 | 0.003 | 0.014 | 0.024 | 0.009 | 0.008 | 0.026 | 0.074 | 0.020 | 0.013 | 0.016 | 0.077 | 0.006 | 0.007 | 0.050 | 0.004 | 0.004 | 0.074 | 0.032 | 0.009 | 0.023 | 0.048 | 0.082 | 0.053 | 0.018 | 0.000 |  |  |  |
| Pima | 0.189 | 0.179 | 0.133 | 0.119 | 0.110 | 0.123 | 0.145 | 0.184 | 0.076 | 0.071 | 0.095 | 0.033 | 0.063 | 0.074 | 0.024 | 0.120 | 0.112 | 0.036 | 0.031 | 0.022 | 0.092 | 0.069 | 0.035 | 0.050 | 0.032 | 0.066 | 0.088 | 0.053 | 0.049 | 0.084 | 0.168 | 0.080 | 0.070 | 0.069 | 0.173 | 0.052 | 0.047 | 0.133 | 0.041 | 0.049 | 0.168 | 0.103 | 0.051 | 0.081 | 0.130 | 0.039 | 0.041 | 0.005 | 0.027 | 0.000 |  |  |
| Surui | 0.041 | 0.037 | 0.033 | 0.016 | 0.041 | 0.031 | 0.024 | 0.041 | 0.009 | 0.010 | 0.017 | 0.025 | 0.013 | 0.010 | 0.021 | 0.037 | 0.018 | 0.011 | 0.039 | 0.015 | 0.013 | 0.006 | 0.022 | 0.013 | 0.015 | 0.010 | 0.017 | 0.005 | 0.019 | 0.009 | 0.035 | 0.014 | 0.011 | 0.007 | 0.037 | 0.018 | 0.004 | 0.023 | 0.015 | 0.015 | 0.035 | 0.015 | 0.008 | 0.009 | 0.031 | 0.120 | 0.075 | 0.064 | 0.021 | 0.060 | 0.000 |  |
| Colombian | 0.139 | 0.130 | 0.093 | 0.079 | 0.077 | 0.086 | 0.102 | 0.135 | 0.042 | 0.039 | 0.064 | 0.006 | 0.029 | 0.040 | 0.005 | 0.080 | 0.075 | 0.016 | 0.005 | 0.006 | 0.055 | 0.040 | 0.008 | 0.025 | 0.015 | 0.038 | 0.053 | 0.026 | 0.026 | 0.052 | 0.120 | 0.046 | 0.035 | 0.038 | 0.125 | 0.021 | 0.021 | 0.089 | 0.016 | 0.018 | 0.120 | 0.063 | 0.025 | 0.051 | 0.086 | 0.053 | 0.046 | 0.008 | 0.006 | 0.017 | 0.038 | 0.000 |
